# The course of pigeon circovirus infection in young pigeons experimentally kept under conditions mimicking the One Loft Race rearing system

**DOI:** 10.2478/jvetres-2025-0009

**Published:** 2025-03-01

**Authors:** Daria Dziewulska, Bartłomiej Tykałowski, Ewa Łukaszuk, Tomasz Stenzel

**Affiliations:** Department of Poultry Diseases, University of Warmia and Mazury in Olsztyn, 10-719 Olsztyn, Poland

**Keywords:** apoptosis, ddPCR, One Loft Race, pigeons, pigeon circovirus

## Abstract

**Introduction:**

Racing pigeon competitions are a popular sport where success depends on birds’ ability to return fast to their loft of origin. However, many additional factors like differences in feeding, training, everyday care and even geographical loft location influence race outcomes, which has led to the development of the One Loft Race (OLR) system. The OLR system aims to eliminate these factors by housing pigeons from various lofts in equal conditions in one facility. This in turn, however, fosters inter-individual transmission of pathogens.

**Material and Methods:**

Fifteen young racing pigeons from five different lofts, naturally infected with pigeon circovirus (PiCV) were reared in one unit for six weeks. Four uninfected birds were kept in a separate unit and were treated as controls for flow cytometry analyses (background establishment). Blood samples were collected every seven days to extract DNA for PiCV quantification using droplet digital PCR and to isolate the mononuclear cells for flow cytometry analyses. On day 42, all birds were euthanised for spleen samples to be collected for further analyses.

**Results:**

The viraemia peak was noted on day 14 of the experiment and subsequently decreased afterwards, with a remarkable decrease noted on day 35. The percentage of IgM^+^ B lymphocytes, including apoptotic cells, in the blood was very similar throughout the experiment. The percentage of apoptotic splenic IgM^+^ B cells was approximately 40% higher in the experimental group than in the control group.

**Conclusion:**

Study results showed that the birds’ adaptation period and the specific immunity they had probably developed hindered PiCV replication. Mild PiCV infection led to a slight increase of B lymphocyte apoptosis in the spleen.

## Introduction

The sport known as pigeon racing is very popular in many countries. In it, pigeons compete with their ability to return to their place of origin (their home loft), being released from a specific location at a noted time and returning to their home lofts over measured distances. In general, the winner is the pigeon that covered the specified distance the fastest. However, since depending on number of pigeon fanciers taking a part in competition, up to several hundred various places with similar but different distances from the release point may be the final destination, the first pigeon returning from the race is not necessary the winner. There are also many other factors influencing race results, including beneficial geographical location of a certain loft (near characteristic points) as well as various methods used to keep and tend to pigeons by different breeders, which have an impact on the pigeons’ conditions. These methods are using special feeding programmes (with different basic feed and various feed additives), doping, including training and health monitoring in the everyday tending by fanciers, and various tricks raising the pigeons’ motivation (7, 8). The multiplicity of these factors has prompted the development of the One Loft Race (OLR) system, which strives to eliminate most of these factors by keeping racing pigeons originating from various fanciers’ lofts in one big rearing facility. This way, their feeding, training and disease prophylaxis as well as race distance from release point to destination are the same, which increases the probability that the race will be won by the pigeon which is objectively the best. Since pigeons become attached to their place of origin over the course of their life, the OLR system can be implemented only with young birds (6–10 weeks old). This is a drawback of the OLR system, because the health, immunity and infection status of young pigeons originating from different flocks varies to a great extent. Because young pigeons do not yet have fully developed immune system, they are prone to infections (11, 13). That is why keeping numerous young birds (from a few hundred to even thousands) originating from different breeding facilities together for a long period creates the perfect conditions for the inter-individual transmission and spread of various pathogens. It results in the high prevalence of various infectious diseases and a high mortality rate in OLR systems.

Pigeon circovirus (PiCV) is very prevalent in the global pigeon population, and its spread could be facilitated by the trade in birds and pigeon racing (14). The role of this virus in pigeon pathology is unclear; however, currently it is believed to be a suppressor of the humoral immune response because it leads to B lymphocyte apoptosis (15, 16). Asymptomatic infections with PiCV are very common in pigeons of all ages (1, 4, 19, 20, 21, 23). This phenomenon promotes its spreading in a pigeon population, also in the case of OLR systems. Moreover, PiCV circulating in a young pigeon population kept in an OLR system can have an immediate synergistic effect on the incidence of other infections (9, 10).

In view of the exacerbatory effect of this recent development in racing pigeon rearing on pigeon health threats in general and PiCV transmission in particular, a study was performed to investigate the potential correlation between viral replication and induction of immunosuppression by B lymphocyte apoptosis in young racing pigeons naturally infected with PiCV and kept under the conditions of the OLR system.

## Material and Methods

### Birds

The experiment was performed on 15 pigeons aged 4–5 weeks, which were bought as 3 birds from each of five private breeding facilities located in the Warmińsko-Mazurskie Voivodeship in Poland. Those facilities had had a documented PiCV infection in the five years prior to the experiment. All the pigeons used for the experiment were clinically healthy and free of pathogenic fungi, bacteria and parasites; they were also positive for pigeon circovirus genetic material in the blood or cloacal swabs.

### Experiment design

After selection, the experimental birds were reared in one unit of the Pavilion of Experimental Infections belonging to the Department of Poultry Diseases in the Faculty of Veterinary Medicine of the University of Warmia and Mazury in Olsztyn (UWM), Poland, for six weeks, which mimicked the conditions of the OLR system and promoted natural cross-infection with PiCV. In addition, four pigeons negative for PiCV genetic material and necessary to the experiment background blood sample collection for flow cytometry analyses were kept in a separate unit. During the experiment, 1mL blood samples were collected into Vacutainer ethylenediaminetetraacetic acid tubes (BD, Franklin Lakes, NJ, USA) from each bird every 7 d. The blood samples were divided into two parts: (A) 20 μL used for DNA extraction to quantify PiCV viral loads, and (B) 500 μL used for the isolation of mononuclear cells for flow cytometry analyses. The B part was used for evaluation of the percentage of apoptotic and early necrotic cells in the B IgM^+^ lymphocyte subpopulation. On day 42 of the experiment, all birds were euthanised to enable the collection of their spleen samples for mononuclear cell isolation and flow cytometry analyses.

### DNA extraction

Total DNA extraction was performed with an Extractme Genomic DNA kit (Qiagen, Hilden, Germany) according to the manufacturer’s guidelines. The concentration and purity of eluted DNA were measured with a NanoDrop 2000 spectrophotometer (Thermo Fisher Scientific, Wilmington, DE, USA). The samples were then stored at –20°C until further analysis.

### Evaluation of PiCV viraemia with droplet digital PCR (ddPCR)

A ddPCR was performed in accordance with the protocol developed in previous studies (15, 16). Prior to the analysis, the quantification cycle (Cq) value was determined for each sample with a qPCR according to the method developed by Duchatel *et al*. (2) targeting a 139 bp fragment of the replication-associated gene (*rep*). The reaction mixture was as follows: 10 μL of Power Sybr Green PCR Master Mix (Life Technologies, Carlsbad, CA, USA), respectively 0.1 and 0.25 μL of forward and reverse primers (concentration of 10 μM), 7.65 μL of RNase-free water and 2 μL of eluted DNA. The reaction was carried out in a LightCycler Real-Time PCR System (Roche, Basel, Switzerland) under the following conditions: an initial denaturation step at 95°C for 10 min followed by 40 cycles of three-stage amplification: at 95°C for 15 s, 64°C for 30 s and 72°C for 30 s. Next, all samples with a Cq lower than 22 were diluted with distilled water according the rule that one single tenfold dilution increases the Cq by 3. This step was necessary to avoid the oversaturation of PCR droplets, which could make proper quantification impossible. After this step, the ddPCR reaction mixture was prepared with the following composition: 10 μL of QX200 ddPCR EvaGreen Supermix (Bio-Rad, Hercules, CA, USA), 0.22 μL of 10 μM forward and reverse primers (the same as in qPCR), 2.2 μL of template DNA, and 9.36 μL of nuclease-free water. The reaction mixture was mixed with 70 μL of QX200 Droplet Generation Oil for EvaGreen (Bio-Rad, Hercules, CA, USA) in a QX 200 droplet generator (Bio-Rad). The obtained droplet emulsions (40 μL) were further transferred into a semi-skirted PCR-clean 96-well plate and then heat-sealed with pierceable foil, and finally the PCR amplification was carried out in a C1000 Touch thermal cycler (Bio-Rad, Hercules, CA, USA). The ddPCR conditions were as follows: an initial denaturation step at 95°C for 5 min followed by 40 cycles of 95°C for 30 s, and 64°C for 1 min with a ramp rate of 2°C/s; the next cycle was a final hold performed at 4°C for 5 min. After the thermal cycling, the plate containing the droplets was chilled to room temperature and placed in a QX 200 droplet reader (Bio-Rad) for analysis. All samples were investigated in duplicate. The results were expressed as mean PiCV genome copy number +/– standard deviation per specified volume of the sample on each day of sampling.

### Isolation of mononuclear cells

To isolate mononuclear cells from peripheral blood, 1 mL of blood sample suspended in 1 mL of phosphate buffered saline (PBS) was applied onto Histopaque-1077 gradient medium (Merck, Darmstadt, Germany) and then centrifuged at 450 × *g* and 20°C for 10 min. The resultant buffy coat of mononuclear cells was collected into sterile test tubes, washed twice and suspended in 1 mL of PBS (Merck, Darmstadt, Germany). The absolute lymphocyte count (ALC) and lymphocyte viability were determined for each sample with an automatic Vi-cell XR Cell Viability Analyzer (Beckman Coulter, Brea, CA, USA).

To isolate mononuclear cells from the spleen, samples of organs collected during necropsy were ground mechanically in a manual glass homogeniser in the presence of a cell culture medium. Once a homogenous suspension had been achieved, the whole sample was passed through nylon filters with a mesh diameter of 70 μm (Corning, Corning, NY, USA). The resultant filtrate was centrifuged at 450 × *g* and 20°C for 10 min, and the cell pellet was resuspended in mononuclear cell isolation gradient. The layer of mononuclear cells obtained this way was rinsed twice and suspended in sterile PBS with 5% addition of activated bovine serum (Merck, Darmstadt, Germany). Afterwards, the ALC was determined for each sample with the Vi-cell XR Cell Viability Analyzer (Beckman Coulter, Brea, CA, USA).

### Flow cytometry for the evaluation of IgM^+^ B lymphocytes and lymphocyte apoptosis

Mononuclear cells isolated from blood and spleen samples and counted were stained with specific polyclonal goat anti-chicken antibodies (fluorescein isothiocyanate (FITC)- conjugated anti-B IgM; Bio-Rad, Hercules, CA, USA). Approximately 500,000 mononuclear cells isolated from each sample were stained and incubated in darkness on ice for 30 min. Then, the cells were rinsed with PBS twice (Merck, Darmstadt, Germany) and centrifuged, and the obtained cell pellets were resuspended in PBS and used for staining for apoptosis evaluation. The staining was performed according to the protocol described by Stenzel *et al*. (16). Cells stained for extracellular markers were washed on ice with Annexin V binding buffer (BD Biosciences, Franklin Lakes, NJ, USA). The supernatant was removed by centrifugation, and the cells were suspended in the same buffer. Next, the FITC-conjugated Annexin V (BD Biosciences, Franklin Lakes, NJ, USA) and 7-aminoactinomycin D (BD Biosciences, Franklin Lakes, NJ, USA) were added to the cells. After incubation, the cells were diluted with Annexin V binding buffer and analysed with flow cytometry within 1 h using a fluorescence-activated cell sorting FACS Canto II flow cytometer (BD Biosciences, Franklin Lakes, NJ, USA). Data were acquired in FACSDiva Software 6.1.3. (BD Biosciences, Franklin Lakes, NJ, USA). Cells were analysed and immunophenotyped in FlowJo 7.5.5 software (BD Biosciences, Franklin Lakes, NJ, USA). Data were expressed as mean percentages of specific lymphocyte subpopulations +/– standard deviation.

### Statistical analysis

The significance of differences in the percentage of the subpopulations of IgM^+^ B lymphocytes in blood and spleen between the experimental and control group was analysed using Student’s *t*-test. The significance of differences between the sampling dates in PiCV copy number in the blood was analysed using repeated measures analysis of variance followed by Tukey’s test. Differences were considered significant at the confidence level of 95 (P-value < 0.05). All analyses were performed with Statistica 13.3 software (Statsoft, Cracow, Poland).

## Results

### Pigeon circovirus viraemia

At the beginning of the experiment, the average PiCV genome copy number (gcn) in the blood of the examined pigeons was 342 ± 859. The viraemia peak of 6,054 ±/14,556 PiCV gcn was noted on day 14 of the experiment. Thereafter, viraemia declined, falling to 5,500 and 4,600 ± 11,000 PiCV gcn on days 21 and 28 of the experiment, respectively. A remarkable decrease of viraemia was noted on day 35 of the experiment, and its lowest level of 202 ± 679 PiCV gcn was noted on the last sampling date (Table 1). The differences between sampling dates were found statistically insignificant (P-values of 0.57–1.00).

**Table 1.** The results of pigeon circovirus quantification by droplet digital PCR in blood throughout the experimental period

	Day of sampling						
	0	7	14	21	28	35	42
Mean PiCV viral load	342.60	1,334.8	6,054.07	5,526.86	4,665.00	1,261.54	202.69
SD of PiCV viral load	859.24	3,964.54	14,556.08	11,115.70	10,974.15	4,181.36	679.48

1 SD – standard deviation

### Flow cytometry for the evaluation of IgM^+^ B lymphocytes and lymphocyte apoptosis

The results of the flow cytometry analyses are presented in Tables 2 and 3. As shown in Table 2, the percentage of B IgM^+^ lymphocytes in the population of blood mononuclear cells was very similar throughout the experiment in both experimental and control pigeons and reached 7.46 +/– 0.87 and 6.98 +/– 0.7 on average, respectively. The percentage of apoptotic B IgM^+^ lymphocytes of the analysed subpopulation was also similar and reached 2.12 +/– 0.87 % and 2.55 +/– 1.52 % on average in the experimental and control pigeons, respectively. The differences in results were significant only on day 0 of the experiment (P-value = 0.04). In turn, the average percentage of necrotic lymphocytes of the analysed subpopulation was 0.2 +/– 0.25 and 0.24 +/– 0.32 in the experimental and control pigeons, respectively. The differences were significant on day 14 (P-value = 0.03) and 42 (P-value = 0.02) of the experiment.

**Table 2.** The percentage of B IgM+ lymphocytes, including the apoptotic (APO) and necrotic (NECRO) cells, in the population of blood mononuclear cells of the experimental and control pigeons throughout the experiment

Day of sampling	Group											
	Experimental						Control					
	B IgM+ (%)		B IgM APO (%)		B IgM NECRO (%)		B IgM+ (%)		B IgM APO (%)		B IgM NECRO (%)	
	Mn	SD	Mn	SD	Mn	SD	Mn	SD	Mn	SD	Mn	SD
0	7.73	0.9	1.99^a^	0.64	0.45	0.78	7.17	0.83	3.32^b^	2.1	0.16	0.15
7	7.60	1.28	2.46	1.35	0.22	0.33	7.17	0.83	3.32	2.1	0.16	0.15
14	7.14	0.98	3.50	1.49	0.28^a^	0.22	6.95	1.26	3.69	0.78	0.67^b^	0.48
21	7.39	0.67	1.68	0.44	0.12	0.08	7.04	0.37	1.56	0.58	0.14	0.08
28	7.63	0.82	1.07	0.32	0.08	0.07	6.92	0.70	1.18	0.02	0.02	0.03
35	6.93	0.67	2.10	0.56	0.16	0.17	6.36	0.34	1.67	0.39	0.31	0.16
42	7.70	0.74	2.01	0.63	0.04^a^	0.05	7.27	0.55	3.10	2.70	0.17^b^	0.17

1 Different letters (^a,b^) indicate a statistically significant difference in the analysed parameter between investigated groups (P-value < 0.05) in Student’s *t*-test

**Table 3.** The percentage of B IgM+ lymphocytes, including the apoptotic (APO) and necrotic (NECRO) cells, in the population of spleen mononuclear cells of the experimental and control pigeons on the last day of the experiment

Parameter	Group			
	Experimental		Control	
	Mn	SD	Mn	SD
B IgM+ (%)	11.29	0.60	11.23	1.02
B IgM APO (%)	5.14	2.77	3.62	1.31
B IgM NECRO (%)	0.12	0.09	0.11	0.05

**Fig. 1.**
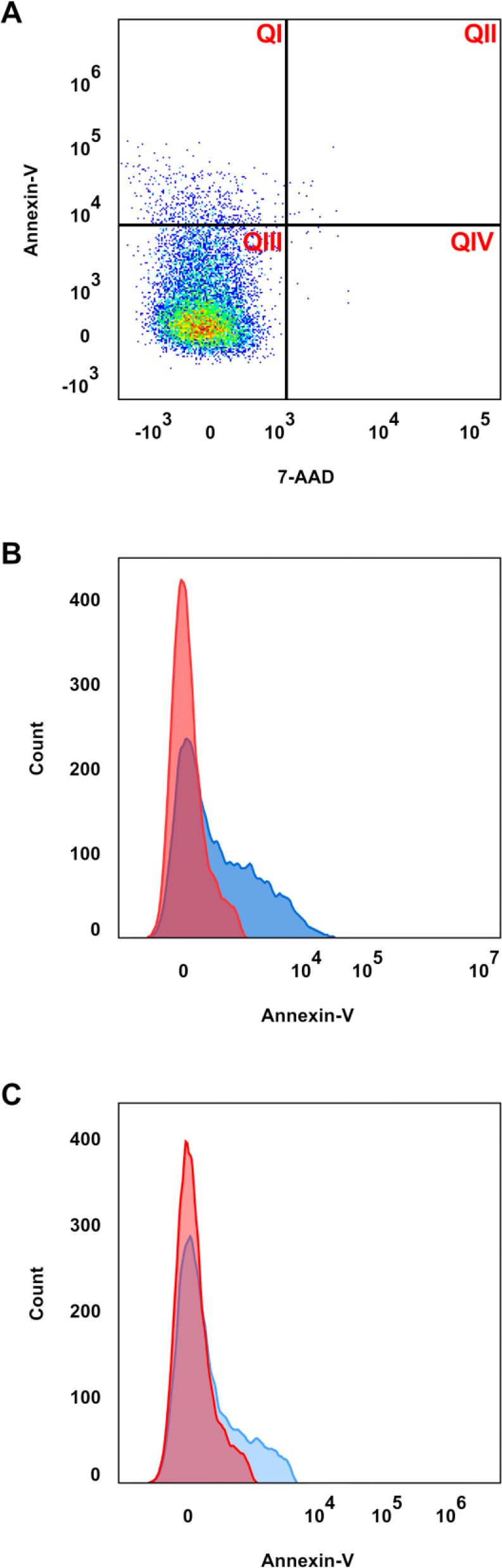
Representative histograms of splenic B IgM^+^ flow cytometry analyses. A. Density plot of the extracellular staining for B IgM, Annexin V and 7-AAD. Three states of the cells were identified: viable cells, QIII (Annexin V/7-AAD = –/–); early apoptotic cells, QIV (Annexin/7-AAD = +/–); necrotic and late apoptotic cells, QI and QII (Annexin V/7-AAD = –/+ and +/+). B. Representative histogram of the sample with high apoptosis. C. Representative histogram of the sample with low apoptosis. The red color on the part B and C indicates on control cells (stained with anti-B IgM antibodies and 7-AAD), whereas the blue color indicates on apoptotic cells stained additionally with Annexin V

As shown in Table 2, there was no statistical difference between the percentages of the individual IgM^+^ B cell subpopulations in the population of splenocytes. The percentage of splenic B IgM^+^ apoptotic cells was ca. 40% higher in the experimental group than in the control one. However, this difference was statistically insignificant (P-value = 0.31). The representative histograms of the analysed blood and spleen samples are shown in Fig. 1.

## Discussion

This experiment was aimed at investigating the potential correlation between the intensity of PiCV infection and the percentage of B lymphocytes, including apoptotic cells. The commercial goat anti-chicken B IgM^+^ polyclonal antibodies were used for the study since they have a proven cross reaction with pigeon lymphocytes (3). PiCV was selected as a viral infection model because it is very prevalent in the global pigeon population and because its infection is rather mild with low mortality (4, 6, 14, 18, 21, 22, 23). Due to the lack of any protocol for laboratory cultivation of PiCV, the young pigeons naturally infected with this virus were used in the experiment (12). OLR has become a popular pigeon sport that can play an important role in pigeon epidemiology by promoting the spreading of various infections and viral recombination (5), which was the reason why the experiment was performed under the conditions mimicking this type of pigeon rearing.

The results of PiCV ddPCR quantification showed that all birds were viremic since the beginning of the experiment, but the virus replication rate was relatively low during the first sampling. The PiCV replication in blood increased successively within the first 14 days of the experiment and peaked in the third sampling date. This increase could be due to the stress of birds during their adaptation to new conditions. The higher viral loads indicate a higher viremia level and/or spreading of PiCV within a flock. After 3 weeks, the viremia level started to gradually decrease and finally fell to almost null on the day of the last sampling. The experiment was performed on pigeons originating from different maternal lofts, hence also differing in their infection and immune status. That is the likely reason of a high standard deviation, which caused the lack of statistically significant differences. On the other hand, the value of standard deviation started to decrease after the infection peak and was the lowest during the last sampling, suggesting PiCV disappearance in all examined birds and equalization of their infectious status. There is not much literature data related to the issues addressed in our research; therefore, the comparative analysis and interpretation of our results is difficult. However, the research conducted by a team of Schmidt *et al*. (12) has shown the highest number of PiCV-positive samples after 14 days post inoculation with a tissue homogenate containing the pigeon circovirus. Moreover, as shown by the study results achieved by this team, the number of PiCV-positive birds successively decreased until the end of the experiment (day 49) (12).

The flow cytometry examination did not provide explicit data. There were no differences in the B IgM^+^ lymphocyte percentage between the pigeons used for the experiment and the control individuals. This observation is inconsistent with findings from our previous study, where a lower percentage of these lymphocytes was determined in pigeons asymptomatically infected with PiCV compared to birds recognized as uninfected (16). The average number of B IgM^+^ cells determined in the blood and spleen in the present study is similar to that reported in one of our previous investigations on the immunogenicity of PiCV recombinant capsid protein (17), but not comparable with that reported by Dziewulska *et al*. (3). However, the cited authors performed an experiment on fantail pigeons (ornamental breed) and not on racing pigeons, and the noted discrepancies could be due to the individual differences between various breeds of pigeons.

An interesting observation was made in the present study, namely: besides small differences (the difference was statistically significant only on the first sampling date), the number of early apoptotic B IgM^+^lymphocytes in the blood was lower in the experimental pigeons compared to control individuals. An opposite trend was noted in the case of lymphocytes isolated from the spleen. This phenomenon indicates differences in general (blood) and local (spleen) response of the lymphocytes to virus replication. In the present study, the number of early apoptotic B IgM^+^ splenocytes was higher in the experimental pigeons, although the difference compared to control birds was not statistically significant. At this point; however, it should be noted that the previous studies have shown a statistically significant increase in apoptotic B lymphocytes only in the pigeons infected with PiCV with evident disease symptoms. Subclinically infected birds did not show a significant increase in the percentage of these cells, which was, however, higher than in the birds recognized as uninfected (16). On this basis, the phenomenon observed in the present investigation should be considered a regularity.

## Conclusion

The results of present study have shown that pigeon circovirus infection can escalate in the conditions of OLR and that PiCV replication is related to the time that has passed since the start of joint rearing. The bird adaptation period and the specific immunity they had probably developed influenced the suppression of the viral replication. Moreover, mild PiCV infection did not lead to significant lymphocyte apoptosis, which makes the theory of pigeon circovirus being their immunosuppressive factor unclear, the same as the role of this virus in pigeon pathology.
